# Niacin exacerbates β cell lipotoxicity in diet-induced obesity mice through upregulation of GPR109A and PPARγ2: Inhibition by incretin drugs

**DOI:** 10.3389/fendo.2022.1057905

**Published:** 2022-12-07

**Authors:** Xiaojing Zhang, Baoyi Zhu, Peibin Lin, Xiaoping Liu, Jun Gao, Dazhong Yin, Jianwen Zeng, Baojian Liao, Zhanfang Kang

**Affiliations:** ^1^ Department of Pharmacy, Qingyuan People’s Hospital, The Sixth Affiliated Hospital of Guangzhou Medical University, Qingyuan, Guangdong, China; ^2^ Department of Urology, Qingyuan People’s Hospital, The Sixth Affiliated Hospital of Guangzhou Medical University, Qingyuan, Guangdong, China; ^3^ Department of Basic Medical Research, Qingyuan People’s Hospital, The Sixth Affiliated Hospital of Guangzhou Medical University, Qingyuan, Guangdong, China; ^4^ Qingyuan People’s Hospital, The Sixth Affiliated Hospital of Guangzhou Medical University, Guangzhou, China; ^5^ Key Laboratory of Regenerative Biology, South China Institute for Stem Cell Biology and Regenerative Medicine, Guangzhou Institutes of Biomedicine and Health, Chinese Academy of Sciences, Guangzhou, China

**Keywords:** niacin, diabetes, incretin drug, β cell, peroxisome proliferator-activated receptor γ (PPAR γ)

## Abstract

The widely used lipid-lowering drug niacin was reported to increase blood glucose in diabetes. How does niacin regulate β Cell function in diabetic patients remains unclear. This study aimed to investigate the effect of niacin on β cell lipotoxicity *in vitro* and *in vivo*. Niacin treatment sensitized the palmitate-induced cytotoxicity and apoptosis in INS-1 cells. In addition, palmitate significantly increased the niacin receptor GPR109A and PPARγ2 levels, which could be further boosted by niacin co-treatment, creating a vicious cycle. In contrast, knocking down of GPR109A could reverse both PPARγ2 expression and niacin toxicity in the INS-1 cells. Interestingly, we found that GLP-1 receptor agonist exendin-4 showed similar inhibitive effects on the GPR109A/PPARγ2 axis and was able to reverse niacin induced lipotoxicity in INS-1 cells. In diet-induced obesity (DIO) mouse model, niacin treatment resulted in elevated blood glucose, impaired glucose tolerance and insulin secretion, accompanied by the change of islets morphology and the decrease of β cell mass. The combination of niacin and DPP-4 inhibitor sitagliptin can improve glucose tolerance, insulin secretion and islet morphology and β cell mass, even better than sitagliptin alone. Our results show that niacin increased β cell lipotoxicity partially through upregulation of GPR109A and PPARγ2, which can be alleviated by incretin drugs. We provide a new mechanism of niacin toxicity, and suggest that the combination of niacin and incretin may have better blood glucose and lipid control effect in clinical practice.

## Introduction

Niacin has been widely used to treat dyslipidemia. Clinical studies have shown that niacin can reduce low-density lipoprotein cholesterol (LDL-C) and triglycerides (TG), and increase high-density lipoprotein cholesterol (HDL-C) levels (1). Therefore, niacin has a potential role in improving cardiovascular risk in patients with dyslipidemia, such as atherosclerosis (2). However, a potentially important side effect of niacin is glycemic regulation, including rising glucose levels in those with diabetes and increasing the risk of developing diabetes in those without diabetes (3–5). Although studies have shown that niacin causes insulin resistance in peripheral tissues, its underlying mechanism is unknown (6). Since the discovery of GPR109A as a niacin receptor in 2003, we have further understood its mechanism, especially its anti-lipolytic effects in adipose tissue (7). GPR109A is expressed in many tissues, such as adipose tissue, pancreas, skin, macrophages, spleen and lung (8).

Recent studies have shown that GPR109A is present in the pancreas β cells and niacin can also act directly on the β cells. In isolated mice islet, niacin inhibits glucose stimulated insulin secretion *via* the G protein-coupled receptor GPR109A pathway (9). Chen et al. reported that niacin induced pancreatic islet dysfunction is probably modulated through activation of the islet beta-cell GPR109a-induced ROS-PPARγ-UCP2 pathways (10). In addition, Yang et al. showed that GPR109A was expressed in mouse MIN6 cell lines and regulated by inflammatory factor INFγ and glucose (11). Niacin receptor GPR109A was also functionally expressed in human islets (12). however the detailed role of β cell GPR109A in the occurrence and development of diabetes needs further study.

At present, obesity and type 2 diabetes are prevalent all over the world. Type 2 diabetes (T2D) is mainly characterized by insulin deficiency, including insulin resistance (insulin relative deficiency) and β Cell insulin secretion decreased (13). Obesity and hyperlipidemia are one of the important factors in the occurrence and development of diabetes. Obesity is associated with elevated concentrations of circulating free fatty acids (FFAs) because of expanded adipose tissue mass and reduced FFA clearance. The deleterious effects of chronically elevated FFA on glucose homeostasis are commonly known as lipotoxicity. Simultaneous exposure to high glucose and high FFA may lead to a synergistic toxic effects called glucolipotoxicity. Lipotoxicity and glucolipotoxicity play an important role in β cell progression dysfunction in type 2 diabetes (14).

Niacin worsens the blood glucose control of pre diabetes and diabetes patients. Though the presence of GPR109A in pancreatic islet cells and the preliminary data on the effects of niacin on insulin secretion and β cell dysfunction have been reported (9, 10), how niacin regulates islet function, especially in diabetes patients, is not fully understood. Since lipotoxicity is an important characteristic of diabetes, in the present study, therefore, we used *in vitro* and *in vivo* approaches to investigate the effect of niacin on β cell lipotoxicity.

## Materials and methods

### Materials and reagents

The INS-1 cell line was obtained from the Chinese Center for Type Culture Collection. Dulbecco′s modified Eagle′s medium (DMEM) and fetal bovine serum (FBS) were purchased from Gibco. Exendin‐4, niacin, sodium palmitate, bovine serum albumin (BSA), and protease inhibitor cocktail were purchased from Sigma‐Aldrich. Sitagliptin was purchased from Hubei Widely Chemical Technology Co., Ltd. Rabbit anti GPR109A was from Bioss. Guinea-pig anti-insulin was from DAKO. Mouse anti-glucagon was purchased from CST. cy2-goat anti-guinea pig and cy3-donkey anti mouse were from Jackson. The PrimeScript™ RT Master Mix kit and the SYBR Green kit were purchased from Takara Biomedical Technology. Triglyceride kit, NEFA (FFA) kit and cholesterol kit were obtained from Wako Company. *In situ* cell death detection kit was purchased from Roche. An 8 mM stock solution of sodium palmitate with 10.5% BSA (the molar ratio of palmitate to BSA was about 5:1) was prepared by dissolving sodium palmitate in DMEM medium as we previously published (15).

### MTT assay

INS-1 cells were cultured in DMEM supplemented with 10% FBS, 100 U/mL penicillin, and 100 μg/mL streptomycin at 37°C in 5% CO2 in air. Before the experiment, cells were seed into 96-well plates with 1.5 × 10^4^ cells/well overnight. The growth medium was then replaced by fresh medium containing different drugs and continue to culture for 24 hours. MTT solution was added into each well with a final concentration of 10% (vol/vol). Then, the cells were incubated at 37°C for 2 hours. Finally, optical densities were measured at 490 nm using a microplate reader.

### RNA extraction and quantitative RT-PCR

Total RNA was extracted using RNAiso Plus according to the manufacturer′s instructions. cDNA synthesis was performed using a PrimeScript RT reagent kit and One-step real-time RT-PCR was performed using SYBR Premix Ex TaqTM according to the manufacturer′s instructions. Primer sequences (Rattus norvegicus) were as follows: GPR109A forward primer, 5’- GAGACAGATGGACAGGCACG-3’; and reverse primer, 5’-TGGTGAAGAAGGCCAAGTCC-3’; PPARγ forward primer, 5’-GGTTATGCGTGTGGGACTCG-3’; and reverse primer, 5’- TGGCTGTTCCATGACTGACC-3’; PPARγ2 forward primer, 5’- CAGGTTTGGGCGAATG-3’; and reverse primer, 5’- TTTGGTCAGCGGGAAG-3’; GAPDH forward primer, 5’- CCTTCCGTGTTCCTACCCC-3’; and reverse primer, 5’- GCCCAGGATGCCCTTTAGT-3’. The data were normalized to the expression levels of GAPDH in each sample.

### Western blot assay

Total protein was extracted with cold RIPA lysis buffer containing protease inhibitor cocktail. Protein levels were measured by BCA kit and separated by SDS-PAGE and transferred to a nitrocellulose (NC) membrane. The protein levels of GPR109A and GAPDH were detected by normal western blot process as described previously (16).

### TUNEL staining analysis β cell apoptosis

INS-1 cells were seeded into Millipore’s Millicell EZ SLIDE and incubated overnight. Next, the cells were treated with different drugs and culture for 24 hours. Then, TUNEL staining kit was used to conduct apoptosis analysis according to the manufacturer’s instructions. Finally, TUNEL-positive cells were observed under a fluorescence microscope. TUNEL positive cells were quantified and expressed as percentage of total cells.

### Small interfering RNA for GPR109A

Three siRNA duplexes targeting rat GPR109A were purchased from Tsingke Biotechnology Co., Ltd. The sequences are as follows: siGPR109A-1 sense, 5’-CGGACAUGAUGACCCGAAA(dT)(dT)-3’, and antisense, 5’- UUUCGGGUCAUCAUGUCCG(dT)(dT)-3’; siGPR109A-2 sense, 5’- GCUGUGGACAGGUACUUCA(dT)(dT)-3’, and antisense, 5’- UGAAGUACCUGUCCACAGC(dT)(dT)-3’; siGPR109A-3 sense, 5’- CGUUCUUGACGGACAACUA(dT)(dT)-3’, and antisense, 5’-UAGUUGUCCGUCAAGAACG(dT)(dT)-3’. GPR109A siRNA or scrambled siRNA was transfected into INS‐1 cells using Lipofectamine RNAiMAX according to the manufacturer′s protocol. After 12 hours of transfection, the cells were treated with different drugs for 48 hours, and finally the cells were collected for corresponding experiments.

### Animals

All animal procedures were performed in accordance with the guidelines for care and use of laboratory animals and approved by The Animal Subjects Committee of Guangzhou Medical University. Male 5 to 6 week-old C57BL/6J mice were obtained from GemPharmatech Co., Ltd. and housed in specific pathogen-free conditions with a 12 hour light-dark cycle and free access to water and food. Mice were fed with chow (Control group) or HFD (Research Diets: D12492, with 60% of kilocalories from fat) for 3 months before the treatment. For drug treatment, sitagliptin (200 mg/kg) was dissolved in 0.5% carboxymethylcellulose and given by gavage once daily; niacin was dissolved in drinking water (10g/L). The mice were administered for 6 weeks. Body weight and fed glucose levels were measured weekly at 9:00 ~ 10:00 a.m. before gavage. 24 hours of food intake were monitored after 4 weeks of treatment by using metabolic cages.

### Glucose tolerance and plasma insulin levels

Glucose tolerance was assessed by oral glucose tolerance test (OGTT) after 4 weeks of treatment. For OGTT, mice were fasted overnight (~17 hours). Glucose levels from tail vein blood were determined immediately using a glucometer (LifeScan, OneTouch Ultra) at 0, 30, 60, and 120 min after an oral administration of 2 g/kg glucose. For plasma insulin determinations, blood was obtained from tail vein by a heparinzed tube 30 min after the oral glucose administration and centrifuged at 7500 rpm at 4°C for 10 minutes, and the supernatant plasma was used for insulin analysis by the Rat/mouse insulin ELISA kit according to the manufacturer’s instructions.

### Insulin tolerance test

ITT was performed after 5 weeks of treatment. For ITT, mice were fasted for 6 hours and intraperitoneally injected with 0.75 IU/kg human insulin. Blood glucose levels were measured in tail vein blood samples that were collected at 0, 30, 60, and 120 min after insulin administration.

### Serum parameters

At the end of experiments, mice were sacrificed and serum was collected and stored at -80 °C before further analysis. Triglycerides (TG), free fatty acid (FFA) and total cholesterol (TC) concentrations were measured using related kits (Wako LabassayTM) according to the manufacturer’s instructions.

### Immunohistochemical analysis of pancreas

Pancreas were quickly dissected from mice and fixed in 4% paraformaldehyde, and paraffin-embedded 4-μm sections were immunostained with guinea-pig anti-insulin and mouse anti-glucagon antibodies overnight at 4°C, followed by staining with cy2-goat anti-guinea pig or cy3-donkey anti-mouse at room temperature for 2 hours. The sample slides were washed three times with 0.1% PBST and stained with DAPI (Invitrogen) before microscopic analysis. β-Cell mass was estimated for each animal by determining the relative β-cell surface area per animal multiplied by their pancreatic weight as previously described (17).

### Statistical analysis

Statistical analyses were performed using GraphPad Prism software. *In vitro* experimental data were expressed as mean ± SD, animal data were presented as mean ± SEM. Differences between the groups were examined for statistical significance using one-way or two-way ANOVA followed by donnett post tests or t tests (as appropriate). P values of less than 0.05 were considered statistically significant.

## Results

### FFA upregulated GPR109A mRNA expression and protein level in INS-1 cells

**Figure 1:**
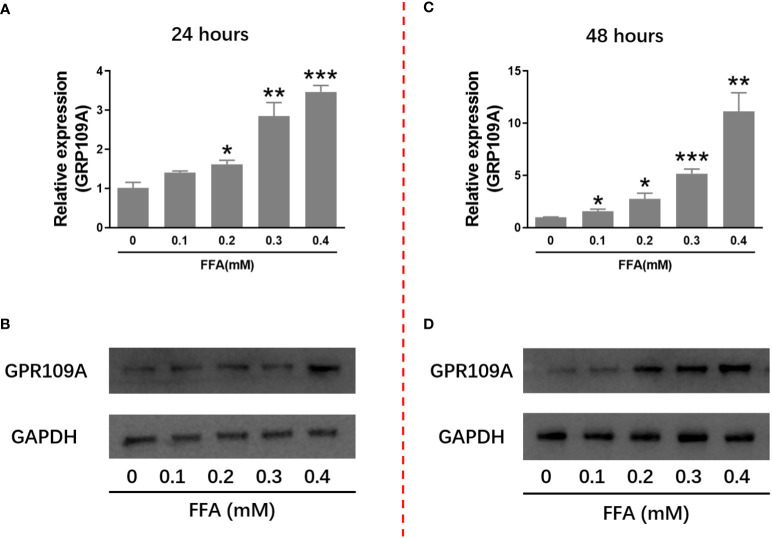
FFA increased GPR109A mRNA expression and protein level in INS-1 cells. INS-1 cells were treated with different concentrations of FFA for 24 or 48 hours. **(A, C)** Total RNA was extracted for quantitative PCR. **(B, D)** Total protein was extracted for western blot analyse. Data were from three independent experiments and represent mean ± SD. n=3, *P < 0.05, **P < 0.01 and ***P < 0.001 compared to the control group (0 mM FFA).

It has been reported that niacin affects β Cell function through its receptor GPR109A. We firstly examined the effect of hyperlipidemia on the expression of GPR109A. We used palmitate *in vitro* to simulate hyperlipidemia *in vivo*. We examined the effect of different concentrations of palmitate (FFA) on GPR109A mRNA expression and protein level. As shown in **Figures 1A–D**, after 24 and 48 hours of 0.1 to 0.4 mM FFA treatment, GPR109A mRNA expression and protein level were significantly increased in a dose - and time-dependent manner.

### Niacin enhances FFA induced cytotoxicity and was alleviated by exendin-4

**Figure 2:**
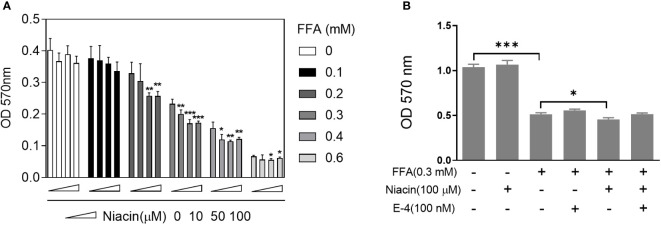
Niacin enhances FFA induced cytotoxicity and was alleviated by exendin-4. After INS-1 cells were treated with different concentrations of niacin and FFA (as indicated) for 24 hours, MTT analysis was performed. **(A)** Niacin dose dependently decreased cell viability in the presence of FFA. **(B)** Exendin-4 restored the cytotoxicity induced by niacin in the presence of FFA. Data were from three independent experiments and represent mean ± SD. *P < 0.05, **P < 0.01 and ***P < 0.001 compared to respective control groups (0 μM niacin).

Since FFA upregulates the expression of niacin receptor GPR109A, we further tested the effect of niacin on INS-1 cytotoxicity in the presence or absence of FFA. As shown in **Figure 2A**, in the absence of FFA or 0.1mM FFA, different concentrations of niacin had no significant effect on the viability of INS-1 cells. Under the condition of 0.2mM FFA, medium and high concentrations of niacin (50 μM and 100 μM) significantly inhibited the viability of INS-1 cells. When the concentration of FFA was 0.3 or 0.4 mM, different concentrations of niacin could inhibit the viability of INS-1 cells. 0.6 mM FFA itself was highly toxic to cells, and the synergistic effect of niacin was not obvious. Because GLP-1 receptor agonists have protective effects on β cells. We further tested the protective effect of the GLP-1 receptor agonist Exendin-4 on niacin and FFA induced cytotoxicity in INS-1 cells. As shown in **Figure 2B**, exendin-4 has no significant effect on 0.3 mM FFA induced cytotoxicity, but it significantly inhibits niacin induced cytotoxicity in the presence of 0.3 mM FFA.

### Niacin increases FFA induced β cell apoptosis and was alleviated by exendin-4

**Figure 3:**
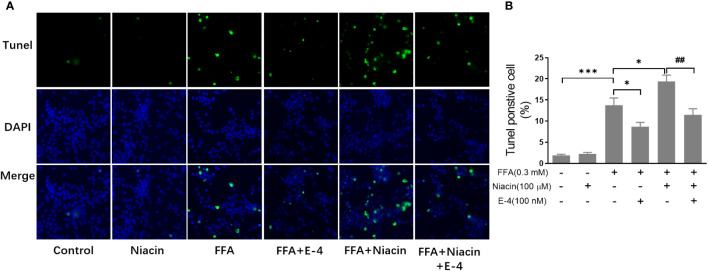
Niacin increases FFA induced β cell apoptosis and was alleviated by exendin-4. INS-1 cells were seeded into Millipore’s Millicell EZ SLIDE and incubated overnight. Next, the cells were treated with niacin (100 μM) or niacin plus exendin-4 (100 nM) in the absence or presence of 0.3 mM FFA for 24 hours. **(A)** Representative images of immunofluorescence. Green, TUNEL positive cells; blue, 4’,6-diamidino-2-phenylindole (DAPI). **(B)** TUNEL positive cells were quantified and expressed as percentage of total cells. The results are displayed as the means ± SD. n=5, *P < 0.05 and ***P < 0.001 compared to the FFA group; ^##^P < 0.01 compared to FFA+Niacin group.

β cell apoptosis plays an important role in the occurrence and development of diabetes. Therefore, we analyzed the apoptosis of INS-1 cells treated with niacin in the presence of FFA. As shown in **Figures 3A, B**, even though 0.3 mm FFA significantly induced apoptosis, the number of apoptotic cells further increased when niacin appeared. As expected, exendin-4 significantly reduced FFA or FFA plus niacin induced apoptosis.

### Niacin up regulates GPR109A and PPARγ2 expressions in the presence of FFA and was inhibited by exendin-4

**Figure 4:**
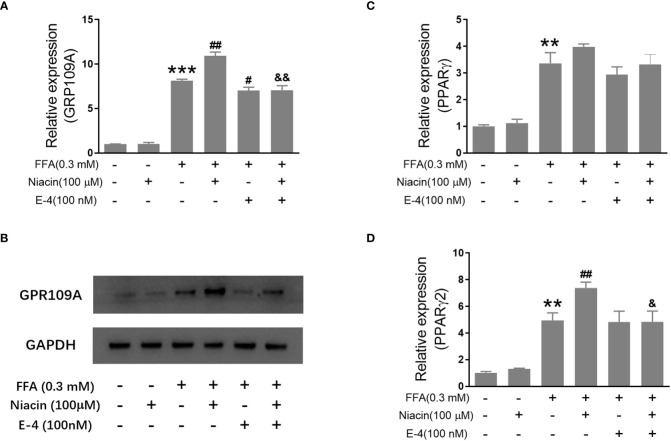
Niacin up regulates the expression of GPR109A and PPARγ2 in the presence of FFA and was inhibited by exendin-4. INS-1 cells were treated with niacin, exendin-4 or niacin plus exendin-4 in the absence or presence of FFA for 48 hours. **(A, C, D)** Total RNA was extracted for quantitative PCR. **(B)** Total protein was extracted for western blot analyse. Data were from three independent experiments and represent mean ± SD. n=3, **P < 0.01 and ***P < 0.001 compared to the control group; ^#^P < 0.05 and ^##^P < 0.01 compared to the FFA group; ^&^< 0.05 and ^&&^< 0.01 compared to the FFA+niacin group.

Studies have shown that niacin receptor and peroxisome proliferator-activated receptor γ (PPARγ) signaling pathway are involved in β Cell function (8, 10). Therefore, we examined the effect of niacin on GPR109A, PPARγ and PPARγ2 expressions under normal and hyperlipidemic conditions. As shown in **Figures 4A, B**, niacin has no effect on the expression of GPR109A mRNA and protein in the normally cultured INS-1 cells in our conditions. In the presence of 0.3mM FFA, niacin further enhanced the expression of GPR109A. Exendin-4 could significantly reduce the expression of GPR109A mRNA and protein level induced by FFA or FFA plus niacin. Accordingly, In the absence of FFA, niacin had no effect on the mRNA expression of PPARγ and PPARγ2 (**Figures 4C, D**). Although FFA significantly increased the mRNA expression of PPARγ and PPARγ2 in INS-1 cells, only PPARγ2 was further increased by niacin in present of FFA. The expression of PPARγ2 induced by FFA plus niacin was also significantly decreased by exendin-4 (**Figures 4C, D**).

### Inhibition of GRR109A mRNA expression by siRNA attenuates niacin induced PPARγ2 expression and lipotoxicity in INS-1 cells

**Figure 5:**
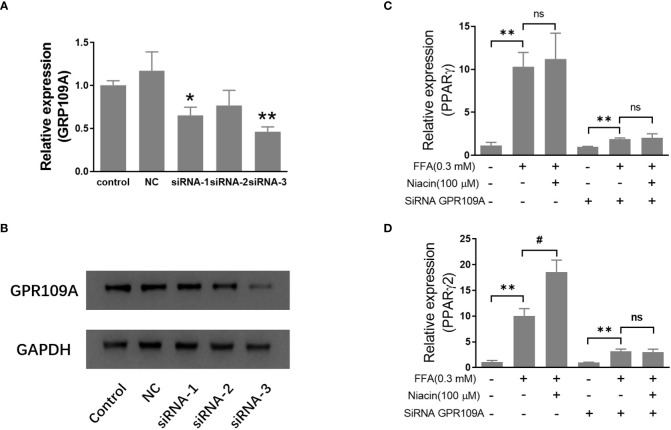
Inhibition of GRR109A mRNA expression by siRNA attenuates niacin induced PPARγ2 mRNA expression in INS-1 cells. INS‐1 cells were transfected with Opti‐MEM (Control), nonspecific siRNA (NC), or three different siRNA (siRNA‐1, siRNA‐2, siRNA‐3) targeting the GPR109A for 12 hours, then cells were treated with 100μM niacin in the absence or presence of 0.3 mM FFA for 48 hours. **(A, C, D)** Total RNA was extracted for quantitative PCR. **(B)** Total protein was extracted for western blot analyse. Data were from three independent experiments and represent mean ± SD. n=3, *P < 0.05 and **P < 0.01 compared to respective control group; ^#^P < 0.05 compared to the siRNA control plus FFA group. NS means not significant (P>0.05).

**Figure 6:**
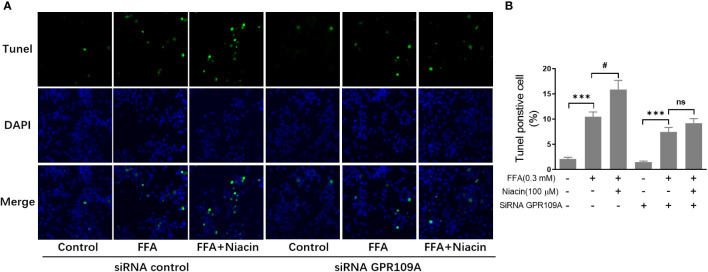
Inhibition of GRR109A mRNA expression by siRNA attenuates niacin induced apoptosis in the presence of FFA. INS‐1 cells were transfected with control or GPR109A siRNA for 12 hours, then cells were treated with FFA or FFA plus niacin for 48 hours. **(A)** Representative images of immunofluorescence. Green, TUNEL positive cells; blue, 4’,6-diamidino-2-phenylindole (DAPI). **(B)** TUNEL positive cells were quantified and expressed as percentage of total cells. The results are displayed as the means ± SD. n = 5, ***P < 0.001 compared to respective control group; ^#^P < 0.05 compared to the siRNA control plus FFA group. NS means not significant (P>0.05).

In order to further investigate the role of GPR109A in the aggravation of lipotoxicity by niacin, we transfected siRNA into INS-1 cells to reduce the expression of GPR109A. We synthesized three siRNAs (siRNA-1, siRNA-2, siRNA-3) and found that siRNA-3 significantly reduced the mRNA expression and protein level of GPR109A (**Figures 5A, B**). Quantitative PCR results showed that FFA significantly increased PPARγ and PPARγ2 gene expression in both control and siRNA groups (**Figures 5C, D**). However, compared with the control group, the expression of PPARγ and PPARγ2 gene induced by FFA was significantly reduced after siRNA transfection, and niacin could not further increase the expression of PPARγ2 gene induced by FFA (**Figures 5C, D**). We further analyzed the effect of siRNA GPR109A on apoptosis in INS-1 cells induced by FFA or FFA plus niacin. As shown in **Figures 6A, B**, the apoptosis induced by FFA in siRNA group tended to decrease, although the statistical difference was not significant. However, siRNA GPR109A significantly inhibited niacin induced apoptosis in the presence of FFA.

### Niacin exacerbates glucose intolerance in diet induced obesity mice and protection by DPP-4 inhibitor sitagliptin

**Figure 7:**
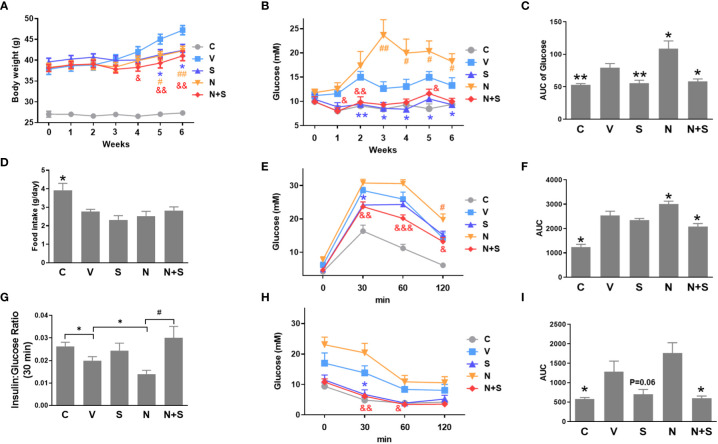
niacin exacerbates glucose intolerance in HFD-induced obesity mice and protection by DPP-4i sitagliptin. HFD mice or normal mice (C group) were treated with vehicle (V group), niacin (N group), sitagliptin (S group) or niacin plus sitagliptin (N+S group) for 6 weeks. Different metabolic indexes were detected during the experiment. **(A)** Body weight, **(B, C)** Random fed blood glucose levels and corresponding area under the curve (AUC). **(D)** 24 hours of food intake. OGTT was carried out after 4 weeks of treatment in mice. **(E–G)** glucose and corresponding AUC and 30-minute insulin and glucose ratio. **(H, I)** ITT results after 4 weeks of treatment. The results are displayed as the means ± SEM. n = 5-9. In **C, D, F, G** and **I**, *P < 0.05 and **P < 0.01 compared to the vehicle group; ^#^P < 0.05 compared to the niacin group. In **A, B, E** and **H**, *P < 0.05 and **P < 0.01 the sitagliptin group compared to the vehicle group; ^#^P < 0.05 and ^##^P < 0.01 the niacin group compared to the vehicle group; ^&^P < 0.05, ^&&^P < 0.01 and ^&&&^P < 0.001 the niacin plus sitagliptin group compared to the vehicle group.

Since our *in vitro* results suggest that niacin aggravates β Cell lipotoxicity and its protection by GLP-1 receptor agonist exendin-4. We next verified whether it was consistent in the animal model of diabetes. In the DIO mouse model, niacin and sitagliptin (DPP-4 inhibitor, an incretin drug) were administered alone or in combination for 6 weeks. As shown in **Figure 7A**, there were no differences between experimental DIO mice groups in body weight before treatment. Compared with the DIO mice control group, the niacin, sitagliptin and niacin plus sitagliptin groups all had significant weight loss in the endpoint of experiment. Fed glucose levels were measured once week during the experiments. As shown in **Figures 7B, C**, compared with mice fed with normal diet, DIO mice treated with vehicle had significantly higher blood glucose levels. Compared with DIO control mice, niacin significantly increased blood glucose, while sitagliptin alone or together with niacin significantly decreased blood glucose (**Figures 7B, C**). However, there were no significantly differences in food intake in DIO mice after treatment with different drugs (**Figure 7D**).

**Figure 8:**
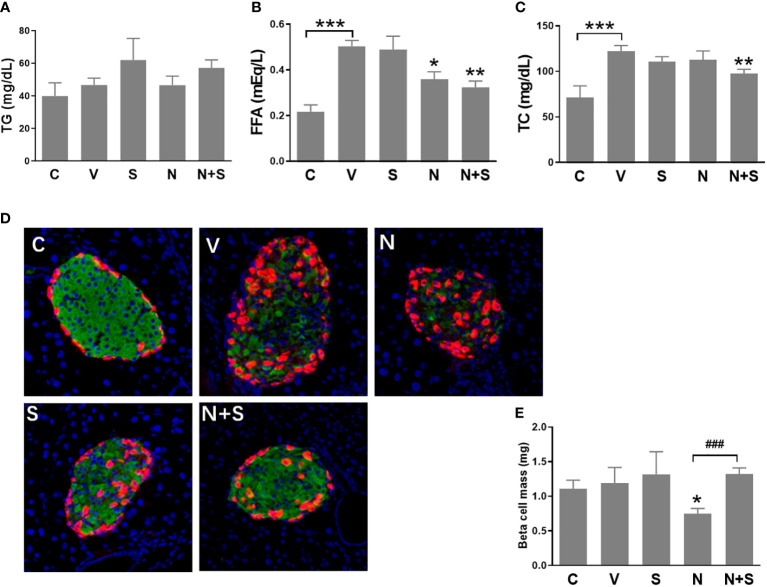
Serum lipid profile and immunohistochemical analysis of the pancreas of the experimental mice. HFD mice or normal mice (C group) were treated with vehicle (V group), niacin (N group), sitagliptin (S group) or niacin plus sitagliptin (N+S group) for 6 weeks. After the experiment, collect serum for biochemical analysis and fix pancreas for immunohistochemical analysis. **(A)** Triglycerides (TG), **(B)** free fatty acids (FFA) and **(C)** total cholesterol (TG). **(D)** Representative images of immunofluorescence analysis of islets stained for insulin (green), glucagon (red) and DAPI (blue). **(E)** Beta cell mass expressed as the percentage of insulin-positive area to total pancreas area. The results are displayed as the means ± SEM. n=5-9, *P < 0.05, **P < 0.01 and ***P < 0.001 compared to the vehicle group; ^###^P < 0.001 compared to the niacin group.

Following the 4-week period of drugs treatment, sensitivity to glucose challenge was assessed by OGTT. As shown in **Figures 7E, F**, compared with the vehicle DIO mice, niacin alone further impaired glucose tolerance, while sitagliptin alone slightly improved glucose tolerance (glucose decreased significantly at 30 min). The combination of niacin and sitagliptin not only inhibited the impairment of glucose tolerance by niacin, but also improved glucose tolerance better than sitagliptin alone. The ratio of insulin to blood glucose at 30 min of oral glucose showed that niacin treatment reduced the ratio, while niacin combined with sitagliptin treatment could return to the normal level (**Figure 7G**). ITT results showed that sitagliptin and sitagliptin plus niacin groups significantly increased insulin sensitivity in DIO mice (**Figures 7H, I**). Serum biochemical indicators showed that there was no significant difference in serum TG level between different experimental groups (**Figure 8A**). Niacin and niacin plus sitagliptin significantly reduced FFA levels (**Figure 8B**). In addition, niacin plus sitagliptin also significantly decreased the serum total cholesterol (TC) level (**Figure 8C**).

### Niacin decreased β cell mass in HFD-induced obesity mice and protection by DPP-4i sitagliptin

We further analyzed the islet morphology and β cell mass in different experimental groups of mice. As shown in **Figure 8D**, the normal islet architecture of control mice comprised a large core of insulin positive β cells ringed by a mantle of glucagon positive α cells. In contrast, islets of vehicle-treated DIO mice exhibited an abnormal architecture with reduced β cells and increased α cells which were gradually distributed to the islet core. Niacin treatment alone further led to the deterioration of pancreatic islet architecture, including the increase of α cells in the islet core. Mice treated with sitagliptin or sitagliptin plus niacin showed an improvement towards normal islet architecture. Consistent with the changes of islet architecture, niacin significantly reduced the β cell mass. Sitagliptin alone has no effect on the β cell mass, but it can restore the decline of β cell mass caused by niacin (**Figure 8E**).

## Discussion

Niacin has been widely used to treat dyslipidemia and reduce the risk of cardiovascular disease, but is known to cause mild increases in blood glucose levels in some diabetic and non-diabetic patients *via* mechanisms that not completely know (4, 5, 18). Niacin induced hyperglycemia is believed to be at least partly due to the induction of peripheral insulin resistance (6). Lipotoxicity caused by obesity/hyperlipidemia is closely related to the occurrence and development of type 2 diabetes. Our study was designed to investigate the effect of niacin on β cells *in vitro* and *in vivo* under lipotoxicity. In INS-1 cells, we found that palmitate treatment significantly increased the mRNA expression and protein level of niacin receptor GPR109A. Accordingly, niacin further aggravated the cytotoxicity and apoptosis induced by palmitate in INS-1 cells. Quantitative PCR results showed that niacin further increased the expression of GPR109A and PPARγ2 in the presence of FFA. Using siRNA to reduce the expression of GPR109A in INS-1 cells showed that PPARγ2 expression was also reduced, while FFA induced cytotoxicity and apoptosis were decreased, and niacin could not further aggravate β cell lipotoxicity. At the same time, we also found that exendin-4, an incretin-based drug, can protect niacin aggravated β cell lipotoxicity. In HFD mice, niacin impairs glucose metabolism and is associated with reduced β cell mass, and this damage can be protected by the DPP-4 inhibitor sitagliptin.

GPR109A is a G-protein-coupled receptor, which is expressed in pancreatic islets, including almost all β Cells and about 40% α Cells (9). Studies have shown that the expression of GPR109A is regulated under physiological and pathological conditions. We found that FFA significantly increased the expression of GPR109A in INS-1 cells, and niacin plus FFA could further increase it. Our experimental results are consistent with those reported by Chen et al. that niacin significantly increased the expression of GPR109A in INS-1E cells and islets of HFD fed mice (10). Li et al. also reported that GPR109A expression in islet β cells increased with age and up-regulated by interferon-γ9. On the other hand, there were reported that high glucose decreased the expression of GPR109A in min6 cells and GPR109A in islet beta-cells was down-regulated dramatically in type 2 diabetic patients as well as in diabetic db/db mice (11, 12). The molecular mechanism leading to the down-regulation of GPR109A in T2DM is puzzling, since inflammatory cytokines and FFA in islets presumably should have stimulated GPR109A expression. In addition, long-term hyperglycemia may stimulate proliferation and de-differentiation of islet beta-cells, leading to the possibility of decreased expression of GPR109A (19).

PPARs are transcription factors that regulate gene expression following ligand activation (20, 21). PPARγ promotes fatty acid uptake, triglyceride formation and storage in lipid droplets, thereby increasing insulin sensitivity and glucose metabolism (20). Systemic administration of PPARγ agonists thiazolidinediones (TZDs) in type 2 diabetes improves glucose homeostasis in human and animal models through improved insulin sensitivity (20). However, evidence that β cell PPARγ directly contributes to the antidiabetic actions of TZDs is less clear. *In vitro* studies in β cell lines or isolated islets have shown both positive and negative effects of PPARγ activation on β cell function and survival (22–26). Two different β-cell-specific-PPARγ knockout mice showed normal glucose metabolism. Among them, Rosen et al. reported that islets from β-cell-specific PPARγ-deficient mice showed hyperplasia on a chew diet and were unable to undergo high fat diet induced hyperplasia (27). Another report showed that inducible β-cell-specific PPARγ knockout mice showed normal glucose metabolism and islet morphology with little to no effect on islet gene expression in animals exposed to a low- or high-fat diet (28). However, recent reports indicate that overexpression of PPARγ2 specifically in pancreatic β Cells impaired insulin signaling and insulin secretion, exacerbated obesity-induced glucose intolerance and reduced β cell mass (29, 30). In this study, we found for the first time that FFA upregulates the expression of GPR109A and PPARγ2 genes in β cells and niacin has an aggravating effect. Our results are consistent with Chen et al’s report that niacin induces β cell dysfunction by activating PGR109A and PPARγ pathways (10). These suggest that PPARγ, especially PPARγ2, plays a negative role in the regulation of β cell lipotoxicity.

Incretin drugs, including GLP-1 receptor agonists and DPP-4 inhibitors (which increase the level of active GLP-1 *in vivo*), are a new type of drugs for the treatment of type 2 diabetes (31, 32). Studies on cells, rodents and humans have shown that GLP-1 receptor agonists have a protective function in β Cells (31, 32). Therefore, we tested whether GLP-1 receptor agonists have protective effect on niacin exacerbated β cell lipotoxicity. As expected, the GLP-1 receptor agonist exendin-4 significantly reduced the apoptosis of INS-1 cells induced by FFA or FFA plus niacin. Our results are consistent with those reported by Li et al. that GLP-1 receptor agonists protect the pancreatic β cells from lipotoxicity induced endoplasmic reticulum stress and apoptosis (33). In the DIO mouse model, the DPP-4 inhibitor sitagliptin significantly improved the metabolic disorder exacerbated by niacin, including impaired glucose metabolism, abnormal islet morphology and decreased β cell mass. Even the combination of sitagliptin and niacin in DIO mice showed better effects on improving glucose metabolism and islet morphology and function than sitagliptin alone. One possible reason is that, as we previously reported, lipotoxicity leads to impaired GLP-1 receptor signaling pathway in pancreatic beta cells, and improvements in lipid control (such as bezafibrate treatment) in mouse models of obesity and diabetes increase the efficacy of incretin-based therapy (15). Niacin, like bezafibrate, is also used for the treatment of hyperlipidemia disorders, which may also improve the therapeutic effect of incretin drugs.

In summary, our results show that niacin increased β cell lipotoxicity partially through upregulation of GPR109A and PPARγ2. Incretin drugs can improve niacin amplified β cell lipotoxicity *in vitro* (exendin-4) and *in vivo* (sitagliptin). These findings suggest that the combination of niacin and incretin drugs may achieve better glucose and lipid control effect in clinical practice.

## Data availability statement

The original contributions presented in the study are included in the article/Supplementary Material. Further inquiries can be directed to the corresponding authors.

## Ethics statement

The animal study was reviewed and approved by the Sixth Affiliated Hospital of Guangzhou Medical University.

## Author contributions

XZ, BZ, PL, XL, JG performed experiments, analyzed the data. DY contributed to the acquisition of data and revision of the article. ZK, BL and JZ conceived and designed the experiments, performed experiments and wrote the manuscript. All authors contributed to the article and approved the submitted version.
